# Inequality in provider and patient-initiated healthcare cancellations during Covid-19

**DOI:** 10.1016/j.ehb.2025.101550

**Published:** 2025-12

**Authors:** Nikita Jacob, Anastasia Arabadzhyan, Panagiotis Kasteridis, Anne Mason, Nigel Rice

**Affiliations:** a Centre for Health Economics, University of York, UK; b Department of Economics and Related Studies and Centre for Health Economics, University of York, UK

**Keywords:** Displaced demand, Unmet need, Inequalities, Covid-19

## Abstract

The Covid-19 pandemic adversely affected access to healthcare raising concerns about worsening health, unmet need and subsequent ‘displaced’ demand. Yet little is known about how this displaced demand was distributed or whether it reflected patient’s decisions to cancel versus provider’s decisions to ration care. Using survey data for England from the UK Household Longitudinal Study, we examine whether planned care continued (or alternative provided), was cancelled by the provider or cancelled by the patient and how these outcomes vary across socio-demographic, clinical, regional and treatment-type characteristics. We estimate weighted multinomial logit models for April-July 2020 (pooled and wave specific), including region and month effects and a region-month Covid-mortality proxy for local NHS strain. Cancellations were overwhelmingly provider-initiated (87% vs 13% patient-initiated). A clear age gradient emerges: provider-initiated cancellations rise with age while patient-initiated cancellations fall; the provider-to-patient cancellation ratio is much higher for those aged 65+ than for younger adults, consistent with providers ‘moving first’ for older adults under capacity constraints. Several groups experienced ‘double jeopardy’ with elevated risks of both provider and patient cancellation: ethnic minority respondents, people in smaller households, urban residents, and those in the North East and Yorkshire and the Humber regions. Because provider cancellations predominated, providers largely determined which treatments continued. Without safeguards, such rationing risks amplified existing inequalities, particularly for double-jeopardy groups. Backlog recovery should protect elective capacity, especially for procedures, and prioritise proactive outreach and flexible scheduling for these groups, whilst reducing patient-side barriers.

## Highlights

• We analyse the demand- and supply-side impacts of the pandemic on planned healthcare in England.
• Reductions in healthcare activity reflect decisions by patients, care providers or both.
• Majority of the cancellations were provider-initiated. Younger adults were more likely to cancel their own care while older adults faced higher risks of provider cancellations.
• Ethnic minority groups, people living in smaller households and urban areas, residents of the North East and Yorkshire and Humber regions were at a greater risk of both (provider and self-initiated) types of cancellations.
• The pandemic widened pre-existing healthcare inequalities leading to higher unmet care needs and subsequent ‘displaced’ demand amongst these groups.

## 1 Introduction

Healthcare systems around the world experienced large reductions in planned hospital activity following the onset of the Covid-19 pandemic. Disruptions to care can lead to worsening health, unmet need, and subsequent rises in demand for healthcare (‘displaced’ demand). In England, reductions in activity reflected decisions by both patients (or their carers) and providers. On the demand side, some patients deferred or avoided care because of infection risk or a desire to ‘protect the NHS’. On the supply-side, hospitals cancelled or postponed elective procedures and treatments to free capacity for Covid-19 patients and meet infection-control constraints.

Understanding who cancels care is critical for policy. Provider-initiated cancellations reflect supply-side rationing under capacity constraints. Patient-initiated cancellations reflect demand-side barriers such as risk avoidance, access costs, and competing responsibilities. When certain groups are at a heightened risk of both types of cancellations, their likelihood of unmet need is compounded. Identifying these ‘double jeopardy’ groups is essential for backlog prioritisation and targeted outreach that does not widen inequalities.

Evidence from the UK and the US has documented disproportionate reductions in utilisation among the most deprived groups, ethnic minorities, women and those with chronic conditions during the pandemic (Warner et al., 2022, Georghiou et al., 2022, Maddock et al., 2021, Shah et al., 2022, Birkmeyer et al., 2020, Topriceanu et al., 2021). Disparities were also patterned by disability status, diagnosis and service type (Kazakova et al., 2022, Bogh et al., 2021, Akobirshoev et al., 2022). Pre-pandemic economics evidence shows persistent gradients in access and outcomes: socio-economically deprived patients face longer emergency department (ED) waits, receive less complex ED care, are less likely to be admitted, and have higher short-term re-attendance or mortality (Turner et al., 2022); waiting-time inequalities are documented for major procedures (Moscelli et al., 2018, Kasteridis et al., 2023); similar patterns arise in Spain, with patients of lower socio-economic status experiencing longer waits and higher surgical cancellations (Bosque-Mercader et al., 2023). More broadly, lower-income individuals have fewer resources and worse health, implying higher healthcare need even under universal coverage (Cookson et al., 2016). Together, this evidence suggests the pandemic is likely to have amplified pre-existing inequalities.

We aim to characterise cancellations during the first pandemic wave through a supply–demand lens, separating provider-initiated from patient-initiated cancellations, identifying which groups faced double jeopardy, and documenting how patterns vary by treatment type and month. Standard administrative sources are not designed for this purpose: they seldom capture planned treatments that have not yet occurred and typically do not indicate who initiated a cancellation. The UK Household Longitudinal Study (UKHLS) Covid-19 modules do both, enabling a descriptive-analytical approach that distinguishes supply-side from demand-side mechanisms.

In the Covid-19 modules, UKHLS respondents report their access to health services during the pandemic and, for those with planned treatment, whose decision it was to cancel or continue. While longitudinal surveys have been used to examine Covid-related disruptions (e.g. Maddock et al., 2021, Di Gessa et al., 2022, Topriceanu et al., 2021), these studies have not identified who cancelled care or whether some groups were at higher risk of both types of cancellation (double jeopardy). UKHLS allows us to address precisely these evidence gaps.

Using UKHLS data for England (April–July 2020), we analyse the probabilities that planned treatment continued, was cancelled by the provider, or was cancelled by the patient. We (i) disentangle supply and demand-side cancellations; (ii) quantify which socio-demographic and clinical groups faced double jeopardy; and (iii) show how patterns differ by treatment type (tests/consultations, operations/procedures, targeted therapies/other). We estimate a probit model for having any planned treatment and a multinomial logit for cancellation outcomes (provider-initiated, patient-initiated, continued/alternative). Specifications use UKHLS Covid cross-sectional weights, cluster standard errors at the individual level, and include region and month indicators, and region–month Covid-mortality tertiles as a proxy for local NHS strain.

Our research highlights several important findings. Provider-initiated cancellations dominated early in the pandemic (substantially outnumbering patient-initiated cancellations). Younger adults were more likely to cancel their own care (‘self cancel’) while older adults faced higher risks of provider cancellations. Ethnic minority groups, people living in smaller households, those in urban areas, and residents of the North East and the Yorkshire & Humber faced double jeopardy. These patterns have direct implications for equitable backlog recovery and for targeting interventions that reduce unmet need without deepening inequalities.

## 2 Data

The UKHLS contains data collected before and during the Covid-19 pandemic. This allows us to study the effect of Covid-19 on displaced demand and to understand if respondents had any treatment planned and if there were cancellations either by providers or by the patient themselves. Details of the data and variables used are set out below.

### 2.1 UKHLS Covid-19 survey

Also known as ‘Understanding Society’, the UKHLS is a broadly representative longitudinal household survey undertaken in the UK each year and contains detailed information on individual and household characteristics (University of Essex and ISER, 2021a). Around 40,000 households were surveyed in wave 1 (2009), including over 100,000 individuals.

During the pandemic, UKHLS was supplemented by additional Covid-19 surveys in the form of short, web-based questionnaires (University of Essex and ISER, 2021b, University of Essex and ISER, 2021c). The first wave of the Covid-19 survey was fielded in April 2020, with monthly waves until July 2020. The survey was then fielded every two months until March 2021, with a final wave in September 2021.^1^

All households who participated in waves 8 or 9 of the main UKHLS survey were potentially eligible to take part in the Covid-19 study. The survey was restricted to individuals aged 16 and above, and excluded those who were unable to give informed consent, people with unknown postal addresses and non-UK residents. A total of 42,330 individuals were invited to participate in the first survey and 17,761 took part (Institute for Social and Economic Research, 2021). In waves 2 to 4 of the Covid-19 surveys, everyone eligible in wave 1 continued to be eligible irrespective of their previous participation in the survey.

The Covid-19 surveys included a one-off series of questions about a January–February 2020 pre-pandemic ‘baseline’. Respondents were then asked about the impact of the pandemic on individuals and families, covering health and well-being, employment and finances. The health modules covered existing health conditions, including chronic conditions and cancer; whether they were on the NHS shielded patient list^2^; whether they were already seeking treatment; and their access to hospital and community care services.

### 2.2 Study sample

We limit our sample to the first four months of the first wave of the pandemic, April 2020 to July 2020, as these capture the period when the majority of changes in response to the pandemic took place. We restrict our analysis to England (39,005 observations) (Table 1).

**Table 1:** Sample selection.

Timeline	Waves	Obs
April 2020	1	17,761
May 2020	2	14,811
June 2020	3	14,123
July 2020	4	13,754

Total		60,449
Exclude Scotland, Wales, Northern Ireland		48,989
Non-missing on controls		39,005

### 2.3 Variables

To analyse the effect of Covid-19 on displaced demand, we look at treatment planned and cancellations by providers and patients. We investigate how these vary across socioeconomic groups such as ethnicity, age, gender, income, employment and education, and the impact on those with a long-term condition or who were on the NHS shielded patient list.

### 2.4 Outcomes

Outcome measures are constructed from core questions in the UKHLS Covid-19 survey and defined as follows:

*Planned treatment:* All respondents were asked if they had any healthcare treatment planned or in progress. The question related to the period either since 1 January 2020 or since the last time a respondent completed the Covid survey. Treatment options included: (i) tests or consultations, (ii) operations or procedures, (iii) targeted therapy such as for cancer (chemotherapy, radiotherapy planned or in progress) and (iv) any other treatments. We combine responses, such that a value of 0 indicates no treatment planned and 1 indicates that the respondent had some type of planned treatment.

*Cancellations:* Respondents who had a treatment planned were asked if their treatment plans had changed in any way. There were four possible responses: (a) Yes, consultations/treatments cancelled or postponed by the NHS; (b) Yes, alternative treatment provided; (c) Yes, I cancelled or postponed treatment; (d) No, treatment continuing as planned. We combine the responses such that a value of 1 indicates that the consultation or treatment was cancelled or postponed by an NHS provider, 2 indicates that the respondent cancelled or postponed their planned treatment themselves, and 3 indicates that an alternative treatment was provided or that the treatment continued as planned.^3^

### 2.5 Covariates

In order to explore potential inequalities in access to care, our models include an array of individual characteristics. Previous studies on disruptions to hospital care during the pandemic provide a rich source of potential explanatory variables. Our models include age group, sex, education, ethnic group, employment status, household size and income, and whether residential location is rural or urban. Information on informal caregiving (whether the respondent looks after or gives special help to someone sick, disabled or elderly in the household) is available only in the July 2020 wave; we therefore include this variable only in a July-wave model presented in the Appendix (Table A.5). We also include binary indicators for whether an individual has at least one long-term health condition^4^ or is clinically vulnerable, i.e. on the NHS shielded patient list (University of Essex and ISER, 2021b). Models also control for English region^5^ and month (wave). In addition, to proxy local pandemic severity (NHS strain), we include monthly region-level Covid-19 mortality tertiles (low/medium/high) alongside the region and month wave indicators.^6^

For respondents with planned care, we classify treatment into four mutually exclusive categories: (i) tests/consultations; (ii) operations/procedures; (iii) targeted therapy (e.g. chemotherapy/radiotherapy); and (iv) other treatment. Cancellation patterns differ by treatment type (Table A.4): targeted therapies are least disrupted, operations/procedures and ‘other treatment’ are most disrupted, and tests/consultations lie in between. Because the targeted-therapy group is very small, especially for patient-initiated cancellations (n = 4), we merge targeted therapy with other treatment for estimation to avoid sparse cells. In analyses of cancellation outcomes (see Section 5), we include indicators for treatment type, using the combined targeted therapy/other category as the reference. Descriptive cross-tabulations by treatment type are reported in Table A.4.

## 3 Empirical approach

We model the binary indicator for having any healthcare treatment planned using probit models. We estimate (i) a pooled model combining April–July and (ii) separate wave-specific models to gauge whether effects persist or diminish over time. The specification is:

(1) $$\mathrm{Pr}\;\left(Y_{it}=1|X_{it},\omega_{t}\right)=\Phi \;\left(\alpha +X_{it}^{⊤}\beta +\omega_{t}^{⊤}\delta \right).$$

where $Y_{it}$ = 1 if respondent i reports planned treatment in wave t (April, May, June, July), and 0 otherwise; $X_{it}$ includes individual and household characteristics and region indicators; $\omega_{t}$ are month (wave) fixed effects; and Φ is the standard Normal CDF.

To analyse cancellation outcomes during the pandemic, we estimate multinomial logit models on the subsample with planned treatment. Let $Y_{it}\in \left\{1,2,3\right\}$ denote the outcome for individual i in wave t: $Y_{it}$ = 1 if the NHS cancelled a scheduled treatment, 2 if the respondent cancelled and 3 if treatment continued or an alternative was provided. We specify:

(2) $$\mathrm{Pr}\;\left(Y_{it}=k|X_{it},\omega_{t}\right)=\Lambda \;\left(\alpha_{k}+X_{it}^{⊤}\beta_{k}+\omega_{t}^{⊤}\delta_{k};\;k\right),\;k\in \left\{1,2,3\right\}.$$

where $X_{it}$ includes individual and household characteristics, region indicators, and treatment-type indicators; $\omega_{t}$ are month (wave) fixed effects; and Λ is the standard logistic CDF.

For the probit models we report average marginal effects. For the multinomial models, we present results as predicted probabilities rather than coefficients or marginal effects, because they are more interpretable as absolute risk and invariant to the (arbitrary) choice of baseline outcome in multinomial logit models. We calculate predicted probabilities for each outcome and wave and for all levels of categorical variables (including their reference categories).^7^ This allows direct comparisons of each predictor’s impact across all outcome categories. All models use the Understanding Society Covid cross-sectional weights. We cluster standard errors at the individual level. This is essential in the pooled regressions, where individuals contribute repeated observations across waves, as it accounts for within-person correlation of residuals. Applying clustering consistently across all models also makes inference more robust by accommodating heteroskedasticity.

**Table 2:** Summary statistics.

Variables	N	Mean	SD	Min	Max
Planned treatment	39 005	0.168	0.374	0	1

Types of treatment planned:					

Tests/consultations	6 710	0.583	0.493	0	1
Operations/procedures	6 710	0.158	0.364	0	1
Targeted therapy/chemo/radiotherapy/other treatment	6 710	0.260	0.439	0	1

Cancellations:					

Provider cancelled treatment	6 710	0.544	0.498	0	1
Patient cancelled or postponed treatment	6 710	0.092	0.289	0	1
Treatment continued as planned/alternative provided	6 710	0.364	0.481	0	1
Age	39 005	50.028	17.752	16	98
Male	39 005	0.474	0.499	0	1
Ethnic minority group	39 005	0.086	0.280	0	1
No/other qualifications	39 005	0.140	0.347	0	1
GCSE	39 005	0.214	0.410	0	1
A-level	39 005	0.215	0.411	0	1
Degree or higher	39 005	0.430	0.495	0	1
Employed	39 005	0.641	0.480	0	1
Household size	39 005	2.781	1.326	1	11
Household income (£)	39 005	2734.191	2387.451	0	32 000
Urban	39 005	0.774	0.418	0	1
Health condition	39 005	0.499	0.500	0	1
NHS shielding list	39 005	0.069	0.253	0	1

NHS usage relates to the study period of April–July 2020. Educational qualifications are the highest level obtained by the respondent. These are weighted statistics.

## 4 Results

Table 2 presents weighted summary statistics for the study period (April–July 2020). Overall, 17% of respondents reported any planned treatment. Among those with planned care (N = 6710), most planned activity involved tests or consultations (58%), followed by operations/procedures (16%) and targeted therapy/other treatment (26%). Within this group, provider-initiated cancellations (54%) were far more common than patient-initiated cancellations (9%); 36% of planned treatments continued as planned or via an alternative. In the full sample (N = 39,005), the average age was 50 (range 16–98), 47% were male, 9% belonged to an ethnic minority group, 8% lived in urban areas, and 64% were employed.^8^ Educational attainment is 14% no/other qualifications, 21% GCSE, 22% A-level, and 43% degree or higher. The average household size was about 3 (range 1–11), and the average monthly household income was £2734 [median income: £2500].^9^ Nearly half of the respondents reported a long-term health condition and 7% were on the NHS shielding list.

*Planned treatment:* Table 3 presents average marginal effects from probit models of having any planned treatment. Column (1) pools April–July while columns (2–5) report wave-specific results. In the pooled model, the strongest predictors are clinical. Reporting a long-term health condition is associated with about 14 percentage points (pp) higher probability of planned treatment, and being on the NHS shielding list adds about 11 pp. Older age also matters: relative to ages 15–29, those 45–64 (over 65) are about 3 pp (4 pp), more likely to have planned treatment. Male and the employed are less likely than female and the unemployed by roughly 2 pp and 5 pp respectively. Lower-income households are slightly more likely to have planned care and education is positively associated with planned treatment. Ethnicity, household size and urban–rural status show no clear pooled associations. London reports higher planned treatment than the North East but no other regional differences are statistically significant. The effects are broadly stable across waves, with no systematic attenuation from April to July.

**Table 3:** Probit models for planned treatment (marginal effects).

Variables	(1)	(2)	(3)	(4)	(5)
	Pooled	April-20	May-20	June-20	July-20
Age 30–44 (Ref: 15–29)	0.009	0.055***	0.012	−0.009	−0.024
	(0.014)	(0.019)	(0.019)	(0.024)	(0.022)
Age 45–64	0.034***	0.051***	0.046***	0.033	0.004
	(0.013)	(0.017)	(0.016)	(0.023)	(0.021)
Age 65 plus	0.044**	0.045*	0.067***	0.048	0.015
	(0.017)	(0.023)	(0.020)	(0.031)	(0.027)
Male	−0.022***	−0.031***	−0.009	−0.019	−0.026**
	(0.008)	(0.011)	(0.010)	(0.012)	(0.011)
Nonwhite (Ref: White)	−0.019	−0.018	−0.003	−0.016	−0.038**
	(0.014)	(0.025)	(0.020)	(0.022)	(0.019)
GCSE (Ref: No quals)	0.004	−0.004	0.003	−0.005	0.020
	(0.014)	(0.024)	(0.020)	(0.021)	(0.020)
A level	0.031**	0.024	0.011	0.051**	0.039*
	(0.015)	(0.024)	(0.019)	(0.023)	(0.021)
Degree and higher	0.037***	0.021	0.041**	0.043**	0.042**
	(0.013)	(0.022)	(0.018)	(0.019)	(0.018)
Employed	−0.051***	−0.061***	−0.040***	−0.043**	−0.056***
	(0.012)	(0.017)	(0.015)	(0.019)	(0.016)
Household size (1–3) (Ref: 4–11)	0.013	0.019	−0.001	0.020	0.017
	(0.009)	(0.012)	(0.012)	(0.015)	(0.014)
Median HH income (Ref: below median income)	−0.022***	−0.012	−0.015	−0.031**	−0.032***
	(0.007)	(0.011)	(0.011)	(0.012)	(0.011)
Urban	−0.008	−0.017	0.009	−0.014	−0.008
	(0.009)	(0.014)	(0.011)	(0.016)	(0.013)
Health condition (Ref: No health condition)	0.139***	0.170***	0.142***	0.132***	0.110***
	(0.008)	(0.011)	(0.011)	(0.011)	(0.011)
NHS shielding list	0.105***	0.151***	0.115***	0.105***	0.048**
	(0.021)	(0.036)	(0.028)	(0.030)	(0.025)
North West (Ref: North East)	0.023	0.026	−0.001	0.039*	0.033
	(0.020)	(0.027)	(0.034)	(0.022)	(0.035)
Yorkshire and Humber	0.014	0.009	0.013	0.024	0.013
	(0.020)	(0.028)	(0.037)	(0.025)	(0.034)
East Midlands	0.011	−0.030	−0.026	0.057*	0.021
	(0.022)	(0.025)	(0.034)	(0.032)	(0.037)
West Midlands	0.016	0.024	−0.021	0.035	0.005
	(0.019)	(0.027)	(0.034)	(0.024)	(0.034)
East of England	0.026	0.029	−0.006	0.052**	0.007
	(0.020)	(0.029)	(0.033)	(0.025)	(0.033)
London	0.044**	0.044	−0.000	0.069**	0.044
	(0.022)	(0.032)	(0.035)	(0.028)	(0.038)
South East	0.033*	0.023	−0.006	0.055**	0.042
	(0.019)	(0.025)	(0.033)	(0.024)	(0.033)
South West	0.016	0.026	−0.012	0.034	−0.004
	(0.020)	(0.028)	(0.034)	(0.023)	(0.033)
Covid-19 deaths: medium (Ref: Covid-19 deaths: low)	−0.001				
	(0.011)				
Covid-19 deaths: high	0.022				
	(0.020)				
May-20 (Ref: Apr-20)	−0.016				
	(0.013)				
Jun-20	−0.005				
	(0.017)				
Jul-20	−0.022				
	(0.021)				
Observations	39,005	10,316	9950	9504	9235
Log-Likelihood Full Model	−16 390	−4631	−4030	−3965	−3685

Robust standard errors in parentheses. Significance levels: *** p < 0.01, ** p < 0.05, * p < 0.1.

*Cancellations:* Table 4 presents predicted probabilities from the pooled multinomial logit for individuals with planned care (April–July).

Age patterns move in opposite directions for the two cancellation types: provider-initiated cancellations rise with age (e.g., 0.41 at ages 15–29 vs. 0.57 at 45–64), while patient-initiated cancellations fall with age (e.g., 0.14 at 15–29 vs. 0.09 at 45–64). Consequently, continuation is highest for younger adults. Females have a higher probability of provider cancellation than males (by 2 pp) but are less likely to cancel their own care (by 1.3 pp), yielding a similar continuation probability overall. For long-term conditions, continuation is essentially identical for those with and without at least one condition. The decomposition shows a slightly lower provider and slightly higher patient cancellation when a condition is present, but both differences are very small. Individuals on the NHS shielding list have slightly lower self-cancellation and slightly higher continuation than others, with minimal difference in provider cancellation. By treatment type, scheduled operations and procedures carry a higher risk of both provider-initiated and patient-initiated cancellation, with correspondingly lower continuation compared to tests and consultations.

Over the four months of the pandemic, the probability of treatment continuation increased substantially from 0.27 in April to 0.54 in July. This was driven by a steady decline in patient-initiated cancellations by 6.1 pp, and a fall in provider cancellations after peaking in May (0.61 in April, 0.62 in May, 0.5 in June, 0.4 in July). These movements mirror the national easing in pandemic pressure across the study period. After absorbing region and month effects, regions in the high mortality tertile show slightly higher continuation than those in the low tertile within the same month, consistent with selective prioritisation of urgent care under greater local pressure.^10^

**Fig. 1:**
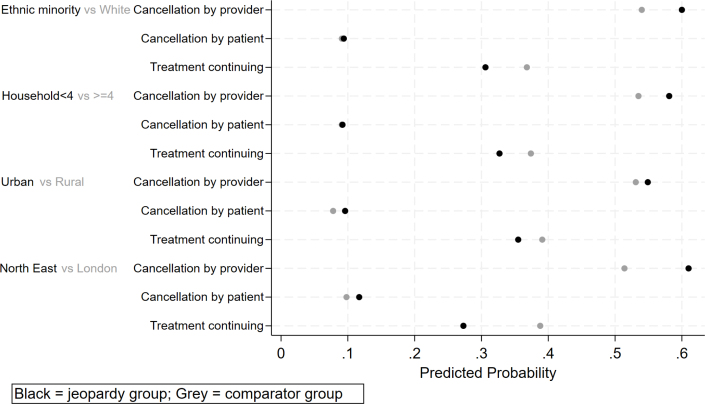
Groups at higher risk of both cancellation types (‘double jeopardy’)

Fig. 1 shows the results for the double jeopardy groups, namely those at higher risk of both NHS-initiated and self-initiated cancellations. Being exposed to both types of disruption placed these groups at a particular disadvantage, as their chances of treatment not continuing were compounded relative to others. Ethnic minorities experience a sharper disadvantage, being 6 pp more likely than people of white ethnicity to face provider cancellations and 0.3 pp more likely to cancel care themselves. Individuals in smaller households are 4.6 pp more likely to face provider cancellations, while the difference in patient-initiated cancellations is relatively small (0.1 pp), compared to those in bigger households. Differences by urban–rural residence are smaller in magnitude, 1.8 pp for both provider and patient cancellations, respectively. Those residing in the North East are 9.6 pp more likely to have their treatment cancelled by the NHS and 1.9 pp more likely to cancel the treatment themselves relative to those from London. Yorkshire and the Humber also shows higher probabilities relative to London (＋6.6 pp provider-initiated; 2.2 pp patient-initiated) placing it in the double jeopardy group (not shown in the figure for compactness, see Table 4). To probe mechanisms behind the household-size pattern, we examined the July wave (the only wave with caregiving data): whereas in pooled models (April–July) respondents in larger households are more likely to continue treatment (Table A.3), in July this association reverses (larger households are more likely to experience disruption), and this holds whether or not we control for caregiving (compare Table A.3, column 4 with Table A.5, column 3). Caregiving itself is strongly associated with patient-initiated cancellations: respondents with caregiving responsibilities are about twice as likely to cancel their own treatment, compared to those without such responsibilities (Table A.5).

**Table 4:** Multinomial model for cancellations (predicted probabilities).

Variables	(1)	(2)	(3)
	Provider cancelled	Self cancelled	Treatment continuing
Age 15–29	0.409***	0.139***	0.453***
	(0.045)	(0.036)	(0.054)
Age 30–44	0.489***	0.113***	0.398***
	(0.031)	(0.022)	(0.032)
Age 45–64	0.574***	0.086***	0.340***
	(0.018)	(0.009)	(0.018)
Age 65 plus	0.573***	0.077***	0.350***
	(0.021)	(0.010)	(0.021)
Female	0.553***	0.086***	0.361***
	(0.014)	(0.007)	(0.015)
Male	0.533***	0.099***	0.368***
	(0.016)	(0.012)	(0.017)
White	0.540***	0.091***	0.368***
	(0.012)	(0.007)	(0.012)
Non-White	0.600***	0.094***	0.306***
	(0.039)	(0.022)	(0.037)
No/other qualification	0.522***	0.082***	0.396***
	(0.036)	(0.019)	(0.038)
GCSE level	0.573***	0.095***	0.332***
	(0.023)	(0.014)	(0.022)
A-level	0.585***	0.074***	0.341***
	(0.023)	(0.011)	(0.023)
Degree or higher	0.522***	0.102***	0.376***
	(0.014)	(0.010)	(0.014)
Unemployed	0.541***	0.107***	0.353***
	(0.019)	(0.012)	(0.020)
Employed	0.547***	0.079***	0.375***
	(0.018)	(0.008)	(0.019)
HH size (1–3)	0.581***	0.092***	0.327***
	(0.025)	(0.013)	(0.024)
HH size (4–11)	0.535***	0.091***	0.374***
	(0.012)	(0.008)	(0.013)
Lower than median HH income	0.550***	0.085***	0.365***
	(0.015)	(0.008)	(0.015)
Equal and above median HH income	0.535***	0.103***	0.362***
	(0.015)	(0.011)	(0.015)
Rural	0.531***	0.078***	0.391***
	(0.022)	(0.011)	(0.023)
Urban	0.549***	0.096***	0.355***
	(0.012)	(0.008)	(0.012)
No- Health condition	0.548***	0.088***	0.364***
	(0.021)	(0.010)	(0.021)
Yes- Health condition	0.543***	0.093***	0.364***
	(0.013)	(0.008)	(0.013)
No- NHS shielding list	0.544***	0.094***	0.362***
	(0.012)	(0.007)	(0.012)
Yes- NHS shielding list	0.547***	0.080***	0.373***
	(0.027)	(0.017)	(0.030)
Targeted therapy/chemo/radiotherapy/other treatment	0.531***	0.138***	0.332***
	(0.020)	(0.014)	(0.020)
Tests/consultations	0.529***	0.071***	0.400***
	(0.014)	(0.008)	(0.014)
Operations/procedures	0.628***	0.086***	0.286***
	(0.022)	(0.015)	(0.022)
North East	0.610***	0.117***	0.273***
	(0.064)	(0.034)	(0.053)
North West	0.560***	0.101***	0.339***
	(0.027)	(0.016)	(0.026)
Yorkshire and Humber	0.580***	0.120***	0.300***
	(0.031)	(0.021)	(0.030)
East Midlands	0.529***	0.097***	0.375***
	(0.037)	(0.025)	(0.036)
West Midlands	0.569***	0.076***	0.355***
	(0.030)	(0.017)	(0.028)
East of England	0.535***	0.064***	0.401***
	(0.029)	(0.015)	(0.031)
London	0.514***	0.098***	0.388***
	(0.043)	(0.022)	(0.047)
South East	0.548***	0.075***	0.377***
	(0.025)	(0.011)	(0.025)
South West	0.500***	0.104***	0.396***
	(0.029)	(0.026)	(0.028)
Covid-19 deaths: low	0.550***	0.108***	0.342***
	(0.037)	(0.027)	(0.030)
Covid-19 deaths: medium	0.540***	0.100***	0.360***
	(0.021)	(0.013)	(0.020)
Covid-19 deaths: high	0.533***	0.077***	0.390***
	(0.037)	(0.014)	(0.037)
Apr-20	0.608***	0.121***	0.272***
	(0.037)	(0.027)	(0.030)
May-20	0.624***	0.100***	0.276***
	(0.022)	(0.014)	(0.020)
Jun-20	0.500***	0.083***	0.417***
	(0.030)	(0.015)	(0.030)
Jul-20	0.398***	0.060***	0.542***
	(0.042)	(0.015)	(0.043)
Observations	6710	6710	6710

Robust standard errors in parentheses. *** p < 0.01, ** p < 0.05, * p < 0.1.

Whilst some effects are pronounced (e.g. those aged 65+ are 17 pp more likely to experience provider cancellations than those under 30), others are modest (e.g. the female-male gap is roughly 2 pp). Even relatively small gaps matter when scaled to the population level, as they imply that systematic disparities affect large numbers of patients.

Results for each outcome across the four waves of the study are shown in the Appendix (Table A.1, Table A.2 and Table A.3).

## 5 Discussion

Previous work shows that the effects of the pandemic on healthcare utilisation varied across clinical and socio-demographic groups (Burn et al., 2021, Propper et al., 2020a, Propper et al., 2020b) raising concerns about unmet need and displaced demand (i.e. care deferred now adding to future need). Distinguishing whether cancellations were provider-led (supply-side) or patient-led (demand-side) is central to backlog recovery and planning resources.

Using detailed survey data that identify who cancelled, we find that several groups were more likely to experience displaced demand. In particular, ethnic minority respondents, people in smaller households and urban residents were more likely both to have treatment cancelled by the NHS and to cancel their own treatment, implying compounded risks of unmet need (i.e. double jeopardy). We also observe heterogeneity by treatment type (Table A.4): operations/procedures faced the highest risks of both provider and patient-initiated cancellation, tests/consultations were less disrupted, and targeted therapies were comparatively protected.

A key feature of the early pandemic is the competing-risk structure: a patient can only cancel if the provider has not already cancelled. Whether a patient would have cancelled care had the provider not cancelled cannot be observed in UKHLS (or any dataset). However, we observe that provider cancellations were around seven times as frequent as patient cancellations, so many appointments never reached a point where a patient choice was possible. This helps explain the age pattern we document: provider-initiated cancellations increase with age, while patient-initiated cancellations decrease with age. Among those aged 65 and over, the provider-to-patient cancellation ratio is markedly higher than for younger adults, indicating that the NHS more often moved first for older adults, leaving fewer opportunities for self-cancellation. These dynamics are consistent with supply-side rationing under capacity constraints to protect those at greater clinical risk.

Patterns over time mirror the easing of early-pandemic pressure. Continuation rises from 0.27 (April) to 0.54 (July), with patient cancellations falling and provider cancellations peaking in May before declining. In pooled regressions that include region and month effects, our proxy for local NHS strain (region–month Covid-mortality tertiles) captures relative severity within a given month. Under that conditioning, higher-severity tertiles are associated with slightly higher probability of continuation, consistent with within-month prioritisation of urgent care in harder-hit areas. In July (the only wave with caregiving information), respondents with caregiving responsibilities are about twice as likely to self-cancel as those without, underscoring that time and care constraints can raise demand-side barriers.

Our interpretation is descriptive rather than causal,^11^ but three features increase confidence that we are observing the amplification of underlying inequalities rather than merely pre-existing differences in care-seeking. First, the timing aligns with the pandemic shock: provider-initiated cancellations (around seven times more frequent than patient cancellations) decline across waves in step with falling Covid mortality. Second, the supply-side dominance (NHS-initiated cancellations far exceeding patient-initiated ones) points to system constraints, not just to baseline patient behaviour. Third, evidence from other care settings shows similar widening of inequalities during the pandemic: domiciliary care studies document rising unmet need and exacerbated inequalities, and outpatient administrative analyses find that pre-existing differences in attendances and cancellations became larger during the pandemic (Arabadzhyan et al., 2025, Kasteridis et al., 2025). Taken together, these points support an interpretation that the pandemic magnified pre-existing inequalities, although we refrain from making strong causal claims.

Our study has some limitations. The analysis covers the first pandemic wave when cancellations were at their highest rates. Therefore, we were unable to investigate ‘what happened next’ in the treatment pathway, and whether the NHS sought to redress imbalances in access by prioritising groups whose access had been most impacted during the early phase of the pandemic. For those who reported that their planned treatment continued or an alternative treatment was provided, we do not know whether the alternative care was appropriate, nor if care was delivered face-to-face or remotely. Instead, we implicitly assume that continued treatment met patients’ need. This assumption may not be valid, and we cannot exclude the possibility that those whose care continued nonetheless experienced unmet need.

Our study demonstrates the importance of understanding the interplay of provider and patient behaviour in order to address and mitigate inequalities in access to care. Future research should exploit longer panels to track the persistence of these patterns, link survey reports to administrative pathways to follow outcomes, and identify where limited resources can most effectively target double-jeopardy groups.

**Table A.1:** Provider cancellations by wave (predicted probabilities).

	Apr-20	May-20	Jun-20	Jul-20
Age 15–29	0.597***	0.517***	0.197***	0.282***
	(0.060)	(0.075)	(0.055)	(0.064)
Age 30–44	0.572***	0.521***	0.463***	0.393***
	(0.046)	(0.055)	(0.052)	(0.055)
Age 45–64	0.660***	0.639***	0.504***	0.454***
	(0.025)	(0.026)	(0.033)	(0.031)
Age 65 plus	0.560***	0.671***	0.596***	0.419***
	(0.036)	(0.029)	(0.038)	(0.032)
Female	0.620***	0.627***	0.501***	0.431***
	(0.022)	(0.021)	(0.026)	(0.024)
Male	0.589***	0.620***	0.504***	0.386***
	(0.025)	(0.025)	(0.028)	(0.026)
White	0.603***	0.626***	0.493***	0.410***
	(0.018)	(0.017)	(0.021)	(0.019)
Non-White	0.650***	0.601***	0.618***	0.434***
	(0.059)	(0.060)	(0.059)	(0.063)
No/other qualification	0.550***	0.654***	0.495***	0.339***
	(0.059)	(0.050)	(0.062)	(0.051)
GCSE level	0.641***	0.684***	0.520***	0.426***
	(0.035)	(0.037)	(0.040)	(0.038)
A-level	0.632***	0.646***	0.566***	0.473***
	(0.037)	(0.038)	(0.043)	(0.043)
Degree or higher	0.601***	0.575***	0.467***	0.406***
	(0.020)	(0.023)	(0.023)	(0.024)
Unemployed	0.628***	0.598***	0.474***	0.432***
	(0.029)	(0.027)	(0.030)	(0.031)
Employed	0.585***	0.650***	0.530***	0.387***
	(0.026)	(0.026)	(0.034)	(0.028)
HH size (1–3)	0.661***	0.635***	0.571***	0.398***
	(0.033)	(0.037)	(0.043)	(0.046)
HH size (4–11)	0.590***	0.621***	0.487***	0.415***
	(0.020)	(0.019)	(0.021)	(0.021)
Lower than median HH income	0.621***	0.608***	0.523***	0.411***
	(0.022)	(0.022)	(0.027)	(0.024)
Equal and above median HH income	0.580***	0.646***	0.473***	0.411***
	(0.024)	(0.025)	(0.026)	(0.026)
Rural	0.596***	0.562***	0.510***	0.410***
	(0.035)	(0.035)	(0.041)	(0.036)
Urban	0.609***	0.641***	0.501***	0.413***
	(0.019)	(0.018)	(0.022)	(0.020)
No- Health condition	0.595***	0.639***	0.520***	0.422***
	(0.032)	(0.031)	(0.035)	(0.035)
Yes- Health condition	0.609***	0.620***	0.497***	0.408***
	(0.019)	(0.020)	(0.022)	(0.021)
No- NHS shielding list	0.596***	0.627***	0.518***	0.403***
	(0.019)	(0.018)	(0.020)	(0.020)
Yes- NHS shielding list	0.659***	0.608***	0.417***	0.464***
	(0.045)	(0.043)	(0.058)	(0.047)
Targeted therapy/chemo/radiotherapy/other treatment	0.550***	0.630***	0.465***	0.447***
	(0.033)	(0.030)	(0.040)	(0.036)
Tests/consultations	0.601***	0.607***	0.492***	0.379***
	(0.022)	(0.022)	(0.023)	(0.023)
Operations/procedures	0.717***	0.680***	0.602***	0.470***
	(0.032)	(0.041)	(0.051)	(0.044)
North East	0.600***	0.701***	0.577***	0.525***
	(0.074)	(0.079)	(0.073)	(0.124)
North West	0.676***	0.607***	0.485***	0.403***
	(0.038)	(0.046)	(0.042)	(0.048)
Yorkshire and Humber	0.649***	0.664***	0.485***	0.470***
	(0.048)	(0.056)	(0.076)	(0.057)
East Midlands	0.616***	0.629***	0.531***	0.297***
	(0.046)	(0.050)	(0.070)	(0.054)
West Midlands	0.586***	0.632***	0.550***	0.474***
	(0.044)	(0.050)	(0.054)	(0.054)
East of England	0.533***	0.665***	0.487***	0.450***
	(0.051)	(0.041)	(0.058)	(0.055)
London	0.518***	0.617***	0.524***	0.411***
	(0.063)	(0.050)	(0.065)	(0.060)
South East	0.656***	0.617***	0.485***	0.400***
	(0.035)	(0.040)	(0.042)	(0.038)
South West	0.609***	0.529***	0.467***	0.355***
	(0.048)	(0.047)	(0.045)	(0.041)
Month	0.606***	0.624***	0.503***	0.412***
	(0.017)	(0.016)	(0.020)	(0.018)
Observations	2004	1663	1593	1450

Robust standard errors in parentheses. Significance levels: *** p < 0.01, ** p < 0.05, *p < 0.1.

**Table A.2:** Patient cancellations by wave (predicted probabilities).

	Apr-20	May-20	Jun-20	Jul-20
Age 15–29	0.114***	0.123***	0.187***	0.122**
	(0.028)	(0.044)	(0.058)	(0.054)
Age 30–44	0.093***	0.172***	0.105***	0.093**
	(0.023)	(0.048)	(0.031)	(0.044)
Age 45–64	0.081***	0.086***	0.106***	0.068***
	(0.012)	(0.015)	(0.022)	(0.014)
Age 65 plus	0.130***	0.085***	0.051***	0.054***
	(0.024)	(0.015)	(0.010)	(0.014)
Female	0.100***	0.101***	0.079***	0.059***
	(0.011)	(0.013)	(0.011)	(0.010)
Male	0.101***	0.100***	0.103***	0.089***
	(0.018)	(0.015)	(0.019)	(0.018)
White	0.099***	0.098***	0.093***	0.068***
	(0.011)	(0.011)	(0.011)	(0.010)
Non-White	0.118***	0.126***	0.056***	0.124**
	(0.032)	(0.040)	(0.018)	(0.059)
No/other qualification	0.105**	0.094***	0.079***	0.049**
	(0.041)	(0.028)	(0.025)	(0.020)
GCSE level	0.127***	0.093***	0.077***	0.080***
	(0.026)	(0.023)	(0.020)	(0.021)
A-level	0.083***	0.074***	0.067***	0.059***
	(0.017)	(0.019)	(0.022)	(0.019)
Degree or higher	0.096***	0.118***	0.109***	0.084***
	(0.011)	(0.017)	(0.017)	(0.017)
Unemployed	0.101***	0.120***	0.114***	0.085***
	(0.016)	(0.019)	(0.019)	(0.020)
Employed	0.099***	0.083***	0.072***	0.059***
	(0.013)	(0.014)	(0.012)	(0.012)
HH size (1–3)	0.106***	0.101***	0.104***	0.054***
	(0.021)	(0.022)	(0.027)	(0.016)
HH size (4–11)	0.099***	0.101***	0.085***	0.078***
	(0.012)	(0.013)	(0.011)	(0.013)
Lower than median HH income	0.093***	0.111***	0.070***	0.061***
	(0.013)	(0.015)	(0.011)	(0.011)
Equal and above median HH income	0.115***	0.087***	0.117***	0.093***
	(0.016)	(0.016)	(0.020)	(0.019)
Rural	0.067***	0.110***	0.071***	0.068***
	(0.014)	(0.022)	(0.017)	(0.018)
Urban	0.112***	0.098***	0.094***	0.073***
	(0.013)	(0.012)	(0.012)	(0.011)
No- Health condition	0.096***	0.136***	0.061***	0.056***
	(0.016)	(0.026)	(0.015)	(0.015)
Yes- Health condition	0.102***	0.090***	0.099***	0.079***
	(0.012)	(0.012)	(0.013)	(0.013)
No- NHS shielding list	0.111***	0.099***	0.085***	0.074***
	(0.013)	(0.011)	(0.011)	(0.010)
Yes- NHS shielding list	0.048***	0.107***	0.110***	0.058**
	(0.016)	(0.029)	(0.026)	(0.023)
Targeted therapy/chemo/radiotherapy/other treatment	0.149***	0.122***	0.149***	0.134***
	(0.021)	(0.019)	(0.028)	(0.024)
Tests/consultations	0.097***	0.079***	0.060***	0.037***
	(0.015)	(0.012)	(0.010)	(0.008)
Operations/procedures	0.034***	0.147***	0.115***	0.086***
	(0.010)	(0.033)	(0.030)	(0.029)
North East	0.104**	0.144***	0.091*	0.107**
	(0.045)	(0.056)	(0.048)	(0.053)
North West	0.118***	0.068***	0.103***	0.087***
	(0.024)	(0.023)	(0.023)	(0.026)
Yorkshire and Humber	0.149***	0.124***	0.071***	0.108***
	(0.038)	(0.034)	(0.024)	(0.036)
East Midlands	0.118***	0.115***	0.095**	0.074**
	(0.036)	(0.040)	(0.043)	(0.037)
West Midlands	0.103***	0.060***	0.084*	0.048**
	(0.027)	(0.019)	(0.044)	(0.019)
East of England	0.066***	0.062***	0.085***	0.050*
	(0.018)	(0.018)	(0.032)	(0.029)
London	0.067***	0.110***	0.126***	0.090***
	(0.019)	(0.036)	(0.033)	(0.031)
South East	0.084***	0.097***	0.069***	0.057***
	(0.019)	(0.023)	(0.019)	(0.016)
South West	0.137**	0.151***	0.069***	0.035**
	(0.054)	(0.040)	(0.019)	(0.017)
Month	0.101***	0.101***	0.089***	0.072***
	(0.010)	(0.010)	(0.010)	(0.010)
Observations	2004	1663	1593	1450

Robust standard errors in parentheses. Significance levels: *** p < 0.01, ** p < 0.05, *p < 0.1.

**Table A.3:** Treatment continuing as planned by wave (predicted probabilities).

	Apr-20	May-20	Jun-20	Jul-20
Age 15–29	0.289***	0.360***	0.616***	0.596***
	(0.057)	(0.070)	(0.076)	(0.070)
Age 30–44	0.336***	0.307***	0.432***	0.514***
	(0.045)	(0.047)	(0.051)	(0.055)
Age 45–64	0.259***	0.275***	0.390***	0.478***
	(0.025)	(0.024)	(0.034)	(0.030)
Age 65 plus	0.310***	0.244***	0.352***	0.527***
	(0.035)	(0.026)	(0.037)	(0.032)
Female	0.279***	0.272***	0.420***	0.510***
	(0.022)	(0.020)	(0.027)	(0.024)
Male	0.310***	0.280***	0.393***	0.525***
	(0.024)	(0.022)	(0.028)	(0.028)
White	0.298***	0.276***	0.414***	0.521***
	(0.017)	(0.015)	(0.021)	(0.019)
Non-White	0.231***	0.273***	0.327***	0.442***
	(0.054)	(0.054)	(0.058)	(0.073)
No/other qualification	0.344***	0.251***	0.426***	0.612***
	(0.059)	(0.044)	(0.067)	(0.051)
GCSE level	0.232***	0.223***	0.403***	0.494***
	(0.029)	(0.033)	(0.039)	(0.039)
A-level	0.285***	0.280***	0.366***	0.468***
	(0.037)	(0.036)	(0.040)	(0.043)
Degree or higher	0.302***	0.307***	0.424***	0.510***
	(0.018)	(0.020)	(0.023)	(0.025)
Unemployed	0.270***	0.283***	0.412***	0.483***
	(0.028)	(0.023)	(0.033)	(0.031)
Employed	0.315***	0.268***	0.398***	0.554***
	(0.026)	(0.023)	(0.035)	(0.029)
HH size (1–3)	0.233***	0.264***	0.325***	0.548***
	(0.029)	(0.033)	(0.041)	(0.049)
HH size (4–11)	0.311***	0.279***	0.429***	0.508***
	(0.019)	(0.017)	(0.022)	(0.020)
Lower than median HH income	0.286***	0.282***	0.407***	0.528***
	(0.021)	(0.020)	(0.027)	(0.024)
Equal and above median HH income	0.305***	0.267***	0.410***	0.497***
	(0.023)	(0.022)	(0.025)	(0.027)
Rural	0.337***	0.328***	0.419***	0.522***
	(0.034)	(0.033)	(0.042)	(0.038)
Urban	0.279***	0.261***	0.405***	0.515***
	(0.018)	(0.016)	(0.022)	(0.020)
No- Health condition	0.308***	0.225***	0.419***	0.521***
	(0.030)	(0.025)	(0.036)	(0.035)
Yes- Health condition	0.288***	0.291***	0.404***	0.514***
	(0.018)	(0.018)	(0.022)	(0.021)
No- NHS shielding list	0.293***	0.274***	0.397***	0.522***
	(0.018)	(0.016)	(0.020)	(0.020)
Yes- NHS shielding list	0.293***	0.285***	0.473***	0.477***
	(0.043)	(0.039)	(0.062)	(0.047)
Targeted therapy/chemo/radiotherapy/other treatment	0.301***	0.247***	0.386***	0.418***
	(0.031)	(0.026)	(0.040)	(0.035)
Tests/consultations	0.302***	0.314***	0.448***	0.584***
	(0.021)	(0.020)	(0.024)	(0.024)
Operations/procedures	0.249***	0.174***	0.283***	0.444***
	(0.031)	(0.035)	(0.048)	(0.045)
North East	0.296***	0.155***	0.332***	0.368***
	(0.065)	(0.057)	(0.063)	(0.104)
North West	0.206***	0.325***	0.412***	0.509***
	(0.031)	(0.045)	(0.041)	(0.051)
Yorkshire and Humber	0.202***	0.212***	0.444***	0.423***
	(0.040)	(0.046)	(0.079)	(0.057)
East Midlands	0.266***	0.256***	0.375***	0.629***
	(0.038)	(0.042)	(0.065)	(0.061)
West Midlands	0.311***	0.308***	0.366***	0.478***
	(0.043)	(0.046)	(0.048)	(0.055)
East of England	0.400***	0.273***	0.428***	0.499***
	(0.055)	(0.037)	(0.060)	(0.053)
London	0.415***	0.274***	0.349***	0.499***
	(0.068)	(0.044)	(0.067)	(0.061)
South East	0.260***	0.286***	0.446***	0.543***
	(0.030)	(0.036)	(0.045)	(0.039)
South West	0.254***	0.320***	0.464***	0.610***
	(0.039)	(0.041)	(0.045)	(0.042)
Month	0.293***	0.276***	0.408***	0.516***
	(0.016)	(0.015)	(0.020)	(0.018)
Observations	2004	1663	1593	1450

Robust standard errors in parentheses. Significance levels: *** p < 0.01, ** p < 0.05, *p < 0.1.

**Table A.4:** Cancellations by type of treatment.

Planned treatment	Cancelled by the provider	Cancelled by patient	Continued as planned	Total
Tests/Consultations	2086	253	1570	3909
	(53.4%)	(6.5%)	(40.2%)	
	{55.8%}	{44.6%}	{65.2%}	{58.3%}
Operations/Procedures	691	77	290	1058
	(65.3%)	(7.3%)	(27.4%)	
	{18.5%}	{13.6%}	{12.0%}	{15.8%}
Targeted therapy/chemotherapy/radiotherapy	67	4	88	159
	(42.1%)	(2.5%)	(55.3%)	
	{1.8%}	{0.7%}	{3.7%}	{2.4%}
Other treatment	892	233	459	1584
	(56.3%)	(14.7%)	(29.0%)	
	{23.9%}	{41.1%}	{19.1%}	{23.6%}
Total	3736	567	2407	6710

Note: Row percentages in parentheses, column percentages in brackets.

**Table A.5:** Multinomial model for Cancellations (Wave 4 controlling for caregiving).

Variables	(1)	(2)	(3)
	Provider cancelled	Self cancelled	Treatment continuing
Age 15–29	0.282***	0.129**	0.590***
	(0.064)	(0.056)	(0.070)
Age 30–44	0.393***	0.095**	0.511***
	(0.055)	(0.045)	(0.054)
Age 45–64	0.452***	0.067***	0.480***
	(0.031)	(0.013)	(0.030)
Age 65 plus	0.420***	0.052***	0.527***
	(0.032)	(0.013)	(0.032)
Female	0.431***	0.059***	0.510***
	(0.024)	(0.010)	(0.024)
Male	0.386***	0.090***	0.524***
	(0.026)	(0.019)	(0.028)
White	0.410***	0.068***	0.521***
	(0.019)	(0.010)	(0.019)
Non-White	0.433***	0.127**	0.440***
	(0.063)	(0.060)	(0.073)
No/other qualification	0.340***	0.052**	0.608***
	(0.051)	(0.021)	(0.051)
GCSE level	0.427***	0.078***	0.495***
	(0.038)	(0.021)	(0.039)
A-level	0.473***	0.057***	0.470***
	(0.043)	(0.019)	(0.043)
Degree or higher	0.406***	0.084***	0.510***
	(0.024)	(0.017)	(0.025)
Unemployed	0.431***	0.083***	0.486***
	(0.031)	(0.020)	(0.031)
Employed	0.388***	0.061***	0.551***
	(0.028)	(0.012)	(0.028)
HH size (1–3)	0.396***	0.051***	0.553***
	(0.047)	(0.016)	(0.049)
HH size (4–11)	0.415***	0.079***	0.506***
	(0.021)	(0.013)	(0.020)
Lower than median HH income	0.411***	0.060***	0.529***
	(0.024)	(0.011)	(0.024)
Equal and above median HH income	0.411***	0.094***	0.495***
	(0.026)	(0.020)	(0.027)
Rural	0.409***	0.067***	0.524***
	(0.036)	(0.017)	(0.037)
Urban	0.413***	0.073***	0.514***
	(0.020)	(0.011)	(0.020)
No- Health condition	0.422***	0.057***	0.521***
	(0.035)	(0.016)	(0.034)
Yes- Health condition	0.408***	0.078***	0.514***
	(0.021)	(0.013)	(0.021)
No- NHS shielding list	0.404***	0.074***	0.522***
	(0.020)	(0.010)	(0.020)
Yes- NHS shielding list	0.464***	0.060**	0.476***
	(0.047)	(0.024)	(0.047)
No- Caregiving responsibilities in house	0.409***	0.066***	0.525***
	(0.019)	(0.010)	(0.019)
Yes- Caregiving responsibilities in house	0.447***	0.136***	0.418***
	(0.058)	(0.039)	(0.057)
Targeted therapy/chemo/radiotherapy/other treatment	0.447***	0.131***	0.423***
	(0.037)	(0.024)	(0.035)
Tests/consultations	0.379***	0.037***	0.584***
	(0.023)	(0.008)	(0.024)
Operations/procedures	0.471***	0.089***	0.440***
	(0.044)	(0.030)	(0.045)
North East	0.527***	0.111**	0.362***
	(0.124)	(0.054)	(0.103)
North West	0.402***	0.083***	0.515***
	(0.048)	(0.025)	(0.050)
Yorkshire and Humber	0.469***	0.113***	0.418***
	(0.057)	(0.037)	(0.056)
East Midlands	0.297***	0.077**	0.625***
	(0.054)	(0.038)	(0.060)
West Midlands	0.475***	0.048**	0.477***
	(0.054)	(0.019)	(0.054)
East of England	0.450***	0.053*	0.498***
	(0.055)	(0.029)	(0.053)
London	0.412***	0.086***	0.502***
	(0.060)	(0.030)	(0.061)
South East	0.401***	0.057***	0.542***
	(0.038)	(0.015)	(0.039)
South West	0.352***	0.034**	0.613***
	(0.041)	(0.016)	(0.043)
Jul-20	0.412***	0.072***	0.516***
	(0.018)	(0.010)	(0.018)
Observations	1450	1450	1450

Robust standard errors in parentheses. *** p < 0.01, ** p < 0.05, * p < 0.1.

## CRediT authorship contribution statement

**Nikita Jacob:** Writing – original draft, Writing – review & editing, Validation, Methodology, Formal analysis, Data curation, Conceptualization. **Anastasia Arabadzhyan:** Writing – review & editing, Visualization, Conceptualization. **Panagiotis Kasteridis:** Writing – review & editing, Visualization, Validation, Formal analysis, Conceptualization. **Anne Mason:** Writing – review & editing, Supervision, Project administration, Funding acquisition, Conceptualization. **Nigel Rice:** Writing – review & editing, Supervision, Funding acquisition, Conceptualization.

## Declaration of competing interest

The authors declare that they have no known competing financial interests or personal relationships that could have appeared to influence the work reported in this paper.

## Data Availability

The authors do not have permission to share data.

## Appendix

See Table A.1, Table A.2, Table A.3, Table A.4, Table A.5.

## Footnotes

1 The Covid-19 surveys are drawn from participants in wave 10 of the UKHLS. To maintain representativeness, Understanding Society provides a cross-sectional weight for each Covid-19 wave. These weights adjust for unequal selection probabilities, differential non-response, and attrition (including deaths, incapacity, and emigration) rescaling respondents back to the weighted wave 10 population. All descriptive and regression analyses in this paper use these weights.

2 The NHS shielded patient list was for people with a clinical condition that put them at high risk of suffering complications from a Covid-19 infection, i.e. they were considered to be clinically extremely vulnerable.

3 Note that the third category combines responses (b) and (d). We group these because both indicate that the patient received treatment, although not necessarily as originally scheduled. The questionnaire does not provide further detail on the equivalence of ‘alternative’ treatment, so it is not possible to assess whether access or quality differed systematically between these responses. Our classification therefore distinguishes between unmet need (cancellations by the NHS or the patient) and met need (treatment delivered, either as planned or in an alternative form).

4 Respondents are asked about a wide range of health conditions including heart disease, respiratory disease, neurological conditions, cancer, mental health, obesity and diabetes.

5 North West, Yorkshire and Humber, East Midlands, West Midlands, East of England, London, South West, South East, North East.

6 The region dummies absorb time-invariant differences across regions, and the month dummies capture shocks common to all regions. The region–month mortality tertiles then identify relative pandemic severity across regions within a given month, and any movements of a region between tertiles across months, i.e., the association between local NHS strain and cancellations net of region and month effects.

7 The baseline category in the multinomial logit is treatment continued as planned or an alternative treatment was provided.

8 In the UKHLS Covid-19 surveys, the non-employed (base group) includes everyone who was not in paid work during January–February 2020.

9 Note that the household income variable has a minimum value of 0. We assume that the values of 0, or slightly above it, correspond to very low income levels, since many respondents reported low incomes and this may represent measurement error. To address this, our analysis uses a categorised income variable which reduces concerns around misreporting and helps to smoothen the impact of the possible errors in measuring household income.

10 When month effects are omitted, the association reverses sign and aligns with the aggregate time pattern: higher mortality coincides with more cancellations.

11 The UKHLS Covid modules ask about planned treatment since 1 January 2020 or since the respondent’s previous Covid survey; cancellation outcomes are conditional on having planned treatment in that reference window. Consequently, month-on-month changes can reflect events reported from the npreceding period (e.g. some April disruptions may be reported in the May wave), and there is no directly comparable pre-pandemic question.
